# Bilateral brucellar breast abscess in a 48-year-old woman

**DOI:** 10.4103/0256-4947.51793

**Published:** 2009

**Authors:** Cem Ibis, Dogan Albayrak, Mehmetali Yagci

**Affiliations:** From the Department of General Surgery, Trakya University Medical Faculty Edirne/Turkey

**To the Editor:**

Brucellosis is a zoonotic infection transmied from animals to humans by ingestion of infected food products, direct contact with an infected animal, or inhalation of aerosols.^1^ The disease remains endemic in many countries, mainly in the Mediterranean basin, the Middle East, India, Mexico, Central and South America and, currently, central and southwest Asia. Soft tissue involvement is an uncommon occurrence of the disease. Breast involvement in animals is common, whereas in humans it is an extremely rare manifestation.^2^–^5^ We report a case of bilateral brucellar breast abscess, which was succesfully treated through abscess drainage and antibiotic therapy.

A 48-year-old woman was admitted to our department with pain, swelling, erythema, and an odorless nipple discharge in both breasts for 10 days. She was involved in stock-breeding on a farm and was suspected of having a history of consumption of unpasteurized cheese. She had also been suffering from night sweats for the previous 2 years. Physical examination revealed a 5×3 cm, indurated, erythematous, and painful mass in the upper left quadrant of the breast on the left side and 3×3 cm periareolar painful mass in the right breast. Bilateral abscess formation with cystic and solid components were detected through ultrasonographic examination of both breasts. Needle puncture of the lesions revealed an obvious purulent material. Bilateral surgical drainage was performed. Oral antibiotic therapy with cefuroxime axetil was began immediately due to a suspected pyogenic abscess. Despite daily wound dressing changes of the abscess cavities and scheduled antibiotic therapy, the patient did not improve.

Approximately 2 weeks after primary abscess drainage, bacterial culture of the purulent material revealed *Brucella* species. Following the positive culture for *Brucella,* the Wright and Rose-Bengal tests were also found to be positive, which confirmed the diagnosis of human brucellosis and bilateral brucellar breast abscess. After replacing the ongoing antibiotic therapy with combined regimen of oral doxycy-cline (200 mg/day) and rifampin (600 mg/day), dramatic regression of both of the abscesses and rapid improvement in clinical symptoms were observed, which also confirmed the diagnosis of human brucellosis. The 6 month follow-up was uneventful.

Brucellar infection of the human breast is extremely rare. Clinical symptoms of brucellosis are nonspesifics and include fever, malaise, sweats, arthralgias, lower back pain, and headache. Based on the series of Andriopoulos et al, the prevalence of human brucellosis as a breast abscess is only 0.7%.^6^ Our case presented with bilateral breast abscesses first though to be pyogenic. Unfortunately, bilateral surgical abscess drainage followed by broad-spectrum oral antibiotic therapy did not heal the abscesses. The delay in the definitive diagnosis of the causative agent of the breast abscess was the most likely reason for failure of treatment. The clinical management of brucellosis is of particular concern because of high initial treatment failure and relapse rates.^7^ Two weeks after initial treatment we were able to replace our initial treatment regimen with appropriate combined antibiotic therapy based on the definitive isolation and positive culture for *Brucella* species as the causative agent in our patient. Although the breast abscess is a very rare complication of systemic brucellosis, the physician should be aware of this unusual manifestation of human brucellosis. We concluded that repeated cultures from purulent material in an unhealing breast abscess, despite the broad-spectrum antibiotic therapy and surgical drainage, may eventually show underlying systemic brucellosis, especially in endemic areas of the world.
